# Appropriateness of clinical severity classification of new WHO childhood pneumonia guidance: a multi-hospital, retrospective, cohort study

**DOI:** 10.1016/S2214-109X(17)30448-5

**Published:** 2017-12-12

**Authors:** Ambrose Agweyu, Richard J Lilford, Mike English, Clinical Information Network Author Group, Grace Irimu, Philip Ayieko, Sam Akech, David Githanga, Fred Were, Barnabas Kigen, Samuel Ng'arng'ar, Nick Aduro, Rachel Inginia, Beatrice Mutai, Grace Ochieng, Lydia Thuranira, Francis Kanyingi, Magdalene Kuria, Sam Otido, Kigondu Rutha, Peris Njiiri, Martin Chabi, Charles Nzioki, Joan Ondere, Caren Emadau, Cecelia Mutiso, Loice Mutai, Christine Manyasi, David Kimutai, Celia Muturi, Agnes Mithamo, Anne Kamunya, Alice Kariuki, Grace Wachira, Melab Musabi, Sande Charo, Naomi Muinga, Mercy Chepkirui, Timothy Tuti, Boniface Makone, Wycliffe Nyachiro, George Mbevi, Thomas Julius, Susan Gachau, Morris Ogero, Michael Bitok, James Wafula

**Affiliations:** a Health Services Unit, KEMRI–Wellcome Trust Research Programme, Nairobi, Kenya; b Nuffield Department of Medicine, University of Oxford, Oxford, UK; c Department of Health Sciences, Warwick Medical School, University of Warwick, Coventry, UK

## Abstract

**Background:**

Management of pneumonia in many low-income and middle-income countries is based on WHO guidelines that classify children according to clinical signs that define thresholds of risk. We aimed to establish whether some children categorised as eligible for outpatient treatment might have a risk of death warranting their treatment in hospital.

**Methods:**

We did a retrospective cohort study of children aged 2–59 months admitted to one of 14 hospitals in Kenya with pneumonia between March 1, 2014, and Feb 29, 2016, before revised WHO pneumonia guidelines were adopted in the country. We modelled associations with inpatient mortality using logistic regression and calculated absolute risks of mortality for presenting clinical features among children who would, as part of revised WHO pneumonia guidelines, be eligible for outpatient treatment (non-severe pneumonia).

**Findings:**

We assessed 16 162 children who were admitted to hospital in this period. 832 (5%) of 16 031 children died. Among groups defined according to new WHO guidelines, 321 (3%) of 11 788 patients with non-severe pneumonia died compared with 488 (14%) of 3434 patients with severe pneumonia. Three characteristics were strongly associated with death of children retrospectively classified as having non-severe pneumonia: severe pallor (adjusted risk ratio 5·9, 95% CI 5·1–6·8), mild to moderate pallor (3·4, 3·0–3·8), and weight-for-age Z score (WAZ) less than −3 SD (3·8, 3·4–4·3). Additional factors that were independently associated with death were: WAZ less than −2 to −3 SD, age younger than 12 months, lower chest wall indrawing, respiratory rate of 70 breaths per min or more, female sex, admission to hospital in a malaria endemic region, moderate dehydration, and an axillary temperature of 39°C or more.

**Interpretation:**

In settings of high mortality, WAZ less than −3 SD or any degree of pallor among children with non-severe pneumonia was associated with a clinically important risk of death. Our data suggest that admission to hospital should not be denied to children with these signs and we urge clinicians to consider these risk factors in addition to WHO criteria in their decision making.

**Funding:**

Wellcome Trust.

## Introduction

Management of children with pneumonia is based on their anticipated risk of poor outcome. In many low-income and middle-income countries that use WHO case management guidelines, hospital admission is recommended when a child crosses the threshold from a non-severe to a severe pneumonia classification according to revised WHO 2013 definitions (table 1).^1^ Hospital care should allow for prompt identification of signs of clinical deterioration and timely intervention with appropriate investigations, treatment, and supportive care including oxygen, fluids, and feeds. Children admitted to hospital could also benefit from expert review that can detect other causes of illness such as heart disease, which might be misdiagnosed as pneumonia by junior clinicians. WHO's revised classification of pneumonia included a change from three to two severity strata, such that children with lower chest wall indrawing are now classed as having non-severe pneumonia, whereas a child with this sign would have automatically been assigned to the severe category under the previous set of criteria.^1^ Other events that have affected the classification of pneumonia include the widespread availability of conjugate vaccines against *Streptococcus pneumoniae* and *Haemophilus influenzae* type b (Hib);^2^ declining prevalence of vertically acquired HIV;^3^ and a technical update of WHO guidelines for the management of severe acute malnutrition in infants and children, which advises the use of weight-for-height Z score (WHZ) and mid-upper-arm circumference to replace weight-for-age Z score (WAZ) for the diagnosis of severe malnutrition requiring inpatient management.^4^ Although studies suggested that the previous WHO clinical algorithm performed similarly across different geographical locations,^5^ concerns have been raised regarding the applicability of new WHO pneumonia guidelines in high mortality settings.^6^, ^7^

**Table 1:** WHO 2013 guidelines for management of children aged 2–59 months with cough, difficulty breathing, or both

	**Clinical signs**	**Recommended setting for management and antibiotic treatment**
Severe pneumonia	Any one of: oxygen saturation <90%, central cyanosis, severe respiratory distress, inability to drink or breastfeed or vomiting everything, altered consciousness, and convulsions	Inpatient management; benzyl penicillin or ampicillin, and gentamicin; add high dose co-trimoxazole for all infants exposed to or infected with HIV
Non-severe pneumonia	Lower chest wall indrawing or fast breathing (respiratory rate ≥50 breaths per min if aged 2–11 months; ≥40 breaths per min if aged 12–59 months), and without signs of severe pneumonia	Outpatient management; oral amoxicillin; for infants with indrawing exposed to or infected with HIV, treat as severe pneumonia

Adapted with permission from WHO.^1^

In this study, we assess risk factors for death in children treated in hospital with pneumonia in Kenya before implementation of the revised WHO pneumonia guidelines. This analysis allows us to explore outcomes in a population of children for whom admission to hospital would no longer be recommended and investigate additional clinical features that might improve risk assessment.

**Research in context**

**Evidence before this study**

We searched MEDLINE for relevant articles published until Jan 16, 2017, on risk factors for community-acquired pneumonia in children in low-income and middle-income countries by use of the terms “respiratory tract infection” [MeSH terms] OR “pneumonia” AND “(child* OR paediatric OR pediatric)” AND (“mortality OR death”). The search was not restricted by date or language. We retrieved a systematic review of 77 observational studies (198 359 children) on risk factors for death from acute lower respiratory infections in children in low-income and middle-income countries.

**Added value of this study**

Published evidence on risk factors for community-acquired childhood pneumonia comes from studies almost entirely done in the pre-pneumococcal vaccine period. The clinical cause of childhood pneumonia is believed to be changing with increasingly high coverage of conjugate vaccines targeting previously dominant causes of pneumonia. This study—the largest published individual analysis of risk factors for mortality among children admitted to hospital with pneumonia—presents findings from a high mortality setting with a high prevalence of comorbidity. We also present unique data on risk of death for subpopulations of children classified as having non-severe pneumonia, who, under existing guidance, might have a level of risk warranting inpatient care.

**Implications of all the available evidence**

The risk factors for pneumonia mortality include severe pneumonia (previously very severe pneumonia), low weight-for-age Z score (WAZ), female sex, age younger than 12 months, *Pneumocystis carinii* infection, HIV infection, young maternal age, low maternal education, low socioeconomic status, secondhand cigarette smoke exposure, indoor air pollution, childhood immunisation status not up to date, non-attendance of antenatal care, and from our study, mild and moderate pallor, respiratory rate of 70 breaths per min or more, admission to hospital in a malaria endemic region, moderate dehydration, and an axillary temperature of 39°C or above. The risk of death for children classified as having non-severe pneumonia with mild to moderate pallor, severe pallor, or low WAZ is similar to that observed for children with WHO-defined severe pneumonia. In high mortality settings, a need exists to re-examine criteria for admission, and clinicians should treat the WHO severe category as but one factor to consider in clinical decision making.

## Methods

### Study design and participants

We did a retrospective cohort study using data from an established network of 14 purposely selected public hospitals situated in regions of high and low to very low malaria transmission in Kenya.^8^ Inpatient records for all children aged 2–59 months admitted to hospital with pneumonia between March 1, 2014, and Feb 29, 2016, were included. We excluded children with documented comorbidities because these are specifically excluded from the WHO pneumonia case management algorithm. Comorbidities include suspected or confirmed meningitis, HIV exposure or infection, severe acute malnutrition (typically identified by presence of visible severe wasting or oedema, skin changes of kwashiorkor, and mid-upper-arm circumference less than 11·5 cm or WHZ less than −3 SD from WHO reference charts), and chronic cardiorespiratory illnesses. Thus, we studied children in whom a clinical diagnosis of pneumonia was the primary indication for antibiotic therapy. WHO uses the terms pneumonia and severe pneumonia to denote groups requiring different treatments. To avoid confusion, we use the term pneumonia to refer to all children with a clinical diagnosis of pneumonia and the terms non-severe and severe pneumonia to refer to children who are recommended outpatient and inpatient treatment respectively by WHO (table 1).

The participating hospitals are typical of district-level health facilities in Kenya and in sub-Saharan Africa, where diagnostic capacity is low. Pulse oximetry data were not available for over 50% of patients and in some hospitals data were not available at all. In line with Kenyan policy recommendations, patients with uncomplicated pneumonia do not routinely have x-rays. In the few cases in which x-rays are done, no structured tools exist for data capture.

The Hib and pneumococcal conjugate vaccines were introduced to the national routine immunisation schedule in 2001 and January, 2011, respectively. Survey data report over 80% coverage for three doses of these vaccines among children aged 12 months.^9^ We restricted our analysis to children who were born after the introduction of Hib and pneumococcal conjugate vaccines.

Data were extracted from patient records devoid of names and contact details that could identify individuals whose information was collected. Data entry was done following patient discharge from hospital or death; thus, the exercise did not interfere with routine patient care or pose apparent additional risk to patients. Ethics approval for the primary study was obtained from the Kenya Medical Research Institute (KEMRI) National Ethical Review Committee. The Ministry of Health and participating hospitals gave permission for the study.

### Procedures

Methods of collection and cleaning of data in the clinical information network are reported in detail elsewhere.^10^ Clinical data for children admitted to hospitals within the network are captured through structured Paediatric Admission Record forms,^11^ approved by the Ministry of Health, that prompt the clinician with a checklist of fields including patient biodata, information gathered during clinical assessment, admission and discharge diagnoses, treatments, and outcome (survival or death). The network supports one data clerk in each hospital to extract data from medical records, nursing charts, treatment charts, and available laboratory reports each day as children are discharged. Data are abstracted from inpatient records into the primary data collection tool developed in Research Electronic Data Capture (REDCap)^12^ with error checks at point of entry by automated daily review, and with regular external data quality assurance.^10^

### Statistical analysis

Categorical data were tabulated and summarised as proportions, whereas continuous variables were reported as mean (SD) or median (IQR) as appropriate. Univariate associations with mortality were calculated for demographic and clinical characteristics with at least 50% of data available. These variables were subsequently considered for inclusion in multivariable logistic regression models restricted to complete cases. Patients' age and sex were included in multivariable models a priori. Hospital identity was included in models as a random effect, whereas location was grouped as a binary variable on the basis of high versus low prevalence of malaria and included as a fixed effect. Other patient characteristics associated with mortality and a p value of less than 0·2 in univariate analyses were added sequentially, beginning with characteristics with the strongest association from univariate models. Likelihood ratio tests were done on addition of each variable to identify the variables to be retained (Model 1). Subsequently, multiple imputation by chained equations (generating ten imputed datasets) was done under missing at random assumption.^13^ The robustness of this assumption was assessed in sensitivity analyses under the assumption of missingness not at random through use of pattern mixture models (appendix).^14^ To enhance efficiency and minimise bias in the imputation procedure, we included variables for which documentation was complete or almost complete even if they were irrelevant to substantive analysis.^15^ Covariates included in Model 1 were then analysed in models including all patients (Model 2) and restricted to non-severe cases (Model 3) through use of imputed datasets. For ease of clinical interpretation, particularly where risk factors were associated with high mortality, we converted odds ratios from logistic regression models to risk ratios.^16^ Accuracy of the three models was analysed by computing areas under the receiver operating characteristic (ROC) curve. For children with non-severe pneumonia, we calculated crude absolute risks of mortality by individual risk factor status as proportions with associated binomial exact 95% CIs. Subdivision of patients with non-severe pneumonia into groups by status of risk factor (with or without risk factor of interest) enabled us to compare risks of death between patients with and patients without the risk factor, and between those with non-severe pneumonia and an individual risk factor and patients with WHO-defined severe pneumonia. No allowance was made for multiple hypothesis testing. Results showing statistical significance close to p=0·05 should therefore be interpreted with caution. We analysed data with STATA version 12.0. The reporting of this study conforms to STROBE recommendations.^17^

### Role of the funding source

The funder of the study had no role in study design, data collection, data analysis, data interpretation, or writing of the report. The corresponding author had full access to all data and had final responsibility for the decision to submit for publication.

## Results

**Figure 1:**
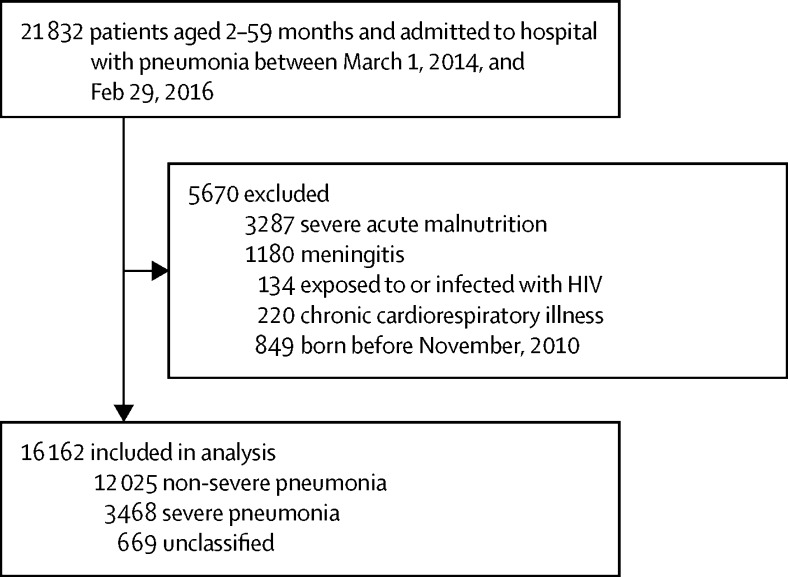
Study profile

21 832 records were available for children aged 2–59 months admitted to any one of 14 hospitals with a diagnosis of pneumonia between March 1, 2014, and Feb 29, 2016. Records for 5875 children with admission diagnoses that would exclude them from the WHO pneumonia case management algorithm were also excluded from the study. Children born before November, 2010, were excluded in keeping with our aim to study children in the post-vaccination era; therefore, analysis included 16 162 children (figure 1).

The median age of the study population was 12 months (IQR 7–24) (table 2). Of 16 127 children for whom sex was documented, 7180 (45%) were girls. 5313 (33%) of 16 162 patients were admitted to hospitals in malaria endemic regions of Kenya. By use of documented clinical diagnosis and available information on clinical signs, we assigned pneumonia severity categories to patients on the basis of the WHO 2013 classification (table 1).^1^ 12 025 (74%) of 16 162 patients had non-severe pneumonia and 3468 (22%) of 16 162 had severe pneumonia. 669 (4%) of 16 162 children had inadequate documentation on key clinical signs required to assign their category of severity. Lower chest wall indrawing was present in 7514 (61%) of 12 323 children, of whom 5320 (71%) were classified with non-severe pneumonia. We excluded children diagnosed with severe acute malnutrition at admission from analysis but calculated WAZ using WHO child growth standards^18^ for children remaining in the cohort because data for these two variables were complete for most patients studied. Although low WAZ might be due to stunting (chronic malnutrition), overlap exists across the categories of severity for mid-upper-arm circumference, WHZ, and WAZ.^19^ Each child was assigned to a nutritional category: normal WAZ (≥–2 SD), low WAZ (<–2 to −3), and very low WAZ (<–3). Data to calculate WAZ were available for 15 330 children of whom 12 324 (80%) had a normal score, 1861 (12%) had a low score, and 1145 (8%) had a very low score—none of the children were assigned a diagnosis of severe acute malnutrition by the admitting clinician. Pallor was present in 1257 (10%) of 12 231 children with available data for the sign (table 2).

**Table 2:** Demographic and clinical characteristics of the study participants

		**Frequency or median**	**Number of patients**
Age (months)		12 (7–22)	16 162
Female		7180 (45%)	16 127
Hospital location: high malaria prevalence*		5313 (33%)	16 162
Immunisation status not up to date†		373 (4%)	9135
Pneumonia severity		··	16 162
	Non-severe	12 025 (74%)	··
	Severe	3468 (21%)	··
	Unclassified	669 (4%)	··
Respiratory rate (breaths per min)		··	10 992
	2–11 months	56 (45–64)	5115
	12–59 months	48 (40–60)	5877
Lower chest wall indrawing present		7514 (61%)	12 323
High fever (axillary temperature ≥39°C)		2049 (18%)	11 706
WAZ		··	15 330
	≥–2 SD (normal)	12 324 (80%)	··
	<–2 SD (low)	1861 (12%)	··
	<–3 SD (very low)	1145 (7%)	··
Pallor		··	12 231
	Absent	10 974 (90%)	··
	Mild to moderate	874 (7%)	··
	Severe	383 (3%)	··
Dehydration		··	16 091
	Absent or no dehydration	14 638 (91%)	··
	Some dehydration	902 (6%)	··
	Severe dehydration	551 (3%)	··

Data are median (IQR) or n (%). WAZ=weight-for-age Z score.

* Versus low to very low malaria prevalence.

† Diphtheria, pertussis, tetanus, *Haemophilus influenzae* type b, hepatitis B, and pneumococcal vaccines: fewer than three doses at 3 months of age or older or fewer than two doses at 2 months of age or older.

Documented outcomes were available for 16 031 (99%) of 16 162 children, and of those, 832 died (5·2%). 488 (14·2%) of 3434 children with severe pneumonia died compared with 322 (2·7%) of 11 930 children within the non-severe category (risk ratio [RR] 5·3, 95% CI 4·6–6·0) (table 3). Mortality was higher in children younger than 12 months than those aged 12–59 months (RR 3·0, 2·6–3·4). Admission to hospitals in regions of high malaria prevalence was associated with increased risk of death (RR 1·3, 95% CI 1·2–1·5). Mortality also increased with decreasing WAZ. Among children with WAZ of −2 SD or more, mortality was 3·8% whereas 7·6% of children with WAZ less than −2 SD to −3 SD and 11·2% of those with scores less than −3 SD died (score test for linear trend p<0·0001). Similarly, mortality increased proportionate to increasing severity of pallor and dehydration. Other characteristics that were associated with increased mortality were elevated respiratory rate (≥70 breaths per min), lower chest wall indrawing, axillary temperature (≥39°C), and female sex. Immunisation status was only weakly associated with mortality (table 3).

**Table 3:** Univariate associations for mortality among all children admitted to hospital with pneumonia

	**Number of deaths/number of patients**	**Mortality (%)**	**RR (95% CI)**	p value*	**P_trend_**
**Pneumonia classification**					
Non-severe	322/11 930	2·7%	1	··	··
Severe	488/3434	14·2%	5·3 (4·6–6·0)	<0·0001	··
Unclassified	22/667	3·3%	1·2 (0·8–1·9)	<0·0001	··
**Age**					
12–59 months	228/8471	2·7%	1	··	··
2–11 months	604/7560	8·0%	3·0 (2·6–3·4)	<0·0001	··
**Sex**					
Male	380/8767	4·3%	1	··	··
Female	447/7115	6·3%	1·4 (1·3–1·7)	<0·0001	··
**Malaria prevalence**					
Low	504/10 747	4·7%	1	··	··
High	328/5284	6·2%	1·3 (1·2–1·5)	<0·0001	··
**Immunisation status**					
Up to date	404/8683	4·7%	1	··	··
Not up to date	27/365	7·4%	1·6 (1·1–2·3)	0·04	··
**Respiratory rate**					
<70 breaths per min	125/1254	4·1%	1	··	··
≥70 breaths per min	394/9657	10·0%	2·4 (2·0–3·0)	<0·0001	··
**Lower chest wall indrawing**					
Absent	132/4770	2·8%	1	··	··
Present	468/7455	6·3%	2·3 (1·9–2·7)	<0·0001	··
**Axillary temperature**					
<39°C	394/9587	4·1%	1	··	··
≥39°C	158/2026	7·8%	1·9 (1·6–2·3)	<0·0001	··
**WAZ**					
≥–2 SD	469/12 235	3·8%	1	··	<0·0001
<–2 to −3 SD	140/1843	7·6%	2·0 (1·6–2·3)	<0·0001	··
<–3 SD	127/1134	11·2%	2·9 (2·4–3·4)	<0·0001	··
**Pallor**					
Absent	396/10 888	3·6%	1	··	<0·0001
Mild to moderate	127/865	14·7%	4·0 (3·3–4·7)	<0·0001	··
Severe	68/382	17·8%	4·8 (3·8–6·0)	<0·0001	··
**Dehydration**					
Absent	635/14526	4·4%	1	··	<0·0001
Some	59/892	6·6%	1·5 (1·2–1·9)	<0·0001	··
Severe	126/543	23·2%	5·2 (4·4–6·0)	<0·0001	··

Weight-for-age Z score (WAZ) based on WHO reference growth standards. Immunisation status omitted in multivariable models. RR=risk ratio.

* p values derived from χ^2^ test.

Adjusted models included all variables analysed in univariate analyses except immunisation status. Parameter estimates from Model 1 (complete case analysis) were similar to those obtained in analyses in which missing data were imputed (table 4). In the full model in which imputed data were used (Model 2), severe pneumonia (*vs* non-severe pneumonia) was independently associated with high mortality (adjusted risk ratio [aRR] 3·9, 95% CI 3·7–4·1). Other characteristics that were strongly associated with mortality (at least double the risk of death) were mild to moderate (aRR 3·4, 95% CI 3·2–3·6) and severe (5·6, 5·1–6·1) pallor, lower chest wall indrawing (2·0, 1·8–2·1), WAZ less than −3 SD (2·1, 1·9–2·2), infants versus children aged 12–59 months (2·5, 2·4–2·7), and children with severe dehydration (2·2, 2·0–2·2) or some dehydration (2·2, 2·0–2·4) versus no dehydration. Female sex, admission to hospital in a region of high malaria prevalence, elevated axillary temperature (≥39°C) or respiratory rate (≥70 breaths per min), and WAZ −2 to −3 SD were all also associated with increased mortality (aRR 1·3–1·9).

**Table 4:** Multivariable models for risk factors for mortality—complete case analysis and multiple imputation analyses (all pneumonia cases and non-severe pneumonia only)

	**Model 1: complete case analysis (all pneumonia cases)**		**Model 2: multiple imputation analysis (all pneumonia cases)**		**Model 3: multiple imputation analysis (non-severe pneumonia only)**	
	Adjusted RR*	95% CI	Adjusted RR*	95% CI	Adjusted RR*	95% CI
**Pneumonia classification**						
Non-severe	1 (ref)	··	1 (ref)	··	··	··
Severe	4·2	(3·4–5·2)	3·9	(3·7–4·1)	··	··
**Age**						
12–59 months	1 (ref)	··	1 (ref)	··	1 (ref)	··
2–11 months	2·7	(2·2–3·4)	2·5	(2·4–2·7)	2·8	(2·6–3·1)
**Sex**						
Male	1 (ref)	··	1 (ref)	··	1 (ref)	··
Female	1·6	(1·3–1·9)	1·5	(1·4–1·6)	1·4	(1·3–1·6)
**Malaria prevalence**						
Low	1 (ref)	··	1 (ref)	··	1 (ref)	··
High	1·3	(1·1–1·7)	1·3	(1·2–1·4)	1·6	(1·4–1·7)
**Respiratory rate**						
<70 breaths per min	1 (ref)	··	1 (ref)	··	1 (ref)	··
≥70 breaths per min	1·8	(1·4–2·3)	1·8	(1·7–1·9)	2·3	(2·1–2·6)
**Lower chest wall indrawing**						
Absent	1 (ref)	··	1 (ref)	··	1 (ref)	··
Present	1·8	(1·4–2·4)	2·0	(1·8–2·1)	2·5	(2·2–2·7)
**Axillary temperature**						
<39°C	1 (ref)	··	1 (ref)	··	1 (ref)	··
≥39°C	1·9	(1·5–2·3)	1·8	(1·7–1·9)	1·9	(1·7–2·1)
**WAZ**						
≥–2 SD	1 (ref)	··	1 (ref)	··	1 (ref)	··
<–2 to −3 SD	1·8	(1·4–2·4)	1·90	(1·8–2·0)	2·4	(2·2–2·7)
<–3 SD	2·1	(1·6–2·9)	2·05	(1·9–2·2)	3·8	(3·4–4·3)
**Pallor**						
Absent	1 (ref)	··	1 (ref)	··	1 (ref)	··
Mild to moderate	3·5	(2·7–4·5)	3·38	(3·2–3·6)	3·4	(3·0–3·8)
Severe	6·1	(4·3–8·7)	5·58	(5·1–6·1)	5·9	(5·1–6·8)
**Dehydration**						
Absent	1 (ref)	··	1 (ref)	··	1 (ref)	··
Some	2·5	(1·7–3·6)	2·17	(2·0–2·4)	2·2	(2·0–2·6)
Severe	2·3	(1·6–3·1)	2·21	(2·0–2·4)	No observations	··

Weight-for-age Z score (WAZ) classification based on WHO reference growth standards. RR=risk ratio.

* Models adjusted for age, sex (a priori covariates), hospital location (fixed effect), immunisation status, WHO pneumonia category (except where only patients with non-severe pneumonia are analysed), respiratory rate, temperature, WAZ, pallor, and dehydration.

We further examined the independent associations for mortality within the category of non-severe pneumonia (for whom outpatient management is recommended) through use of imputed datasets (Model 3). The findings of this analysis were consistent with models using the full datasets (table 4). Areas under the ROC curve for each model showed good accuracy (0·8565, 0·8456, and 0·8115 for model 1, 2, and 3 respectively). Plots from the ROC analysis are provided in the appendix.

**Figure 2:**
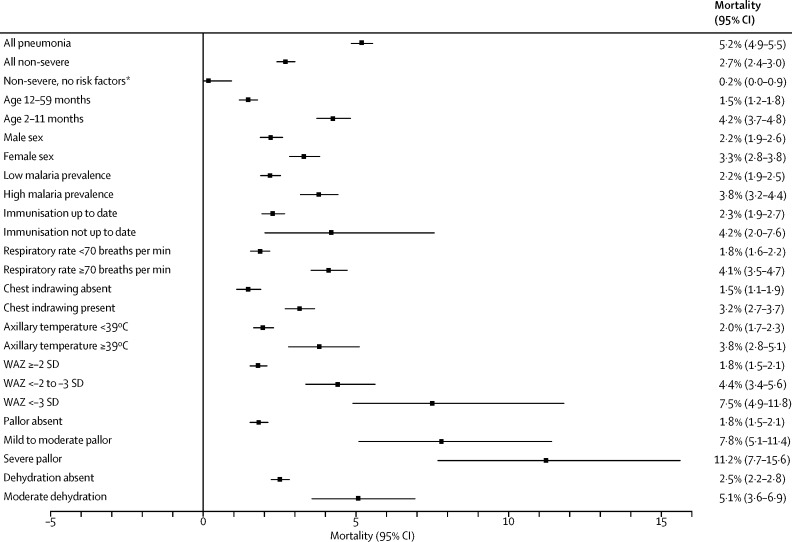
Estimated risks of death among patients with non-severe pneumonia by risk factor status WAZ=weight-for-age Z score. *Excludes patients aged 2–11 months, girls, treatment in hospital in area with high prevalence of malaria, immunisations not up to date, respiratory rate ≥70 breaths per min, chest indrawing present, axillary temperature ≥39°C, WAZ <–2 SD, mild to moderate or severe pallor, or moderate dehydration.

In subgroup analyses confined to children with non-severe pneumonia, estimated mortality of children with WAZ less than −3 SD (7·5%, 95% CI 4·9–11·8), mild to moderate pallor (7·8%, 5·1–11·4), and severe pallor (11·2%, 7·7–15·6) was similar to mortality observed in children with severe pneumonia (upper bound of 95% CI >10%). Risk of death for children with non-severe pneumonia and one risk factor—infants, admission to hospital in a malaria endemic region, respiratory rate of 70 breaths per min or higher, WAZ less than −2 to −3 SD, or moderate dehydration—exceeded the overall risk among children with non-severe pneumonia (lower bound of 95% CI higher than the upper bound for all non-severe pneumonia cases). The absolute risk of mortality among children with non-severe pneumonia and lower chest wall indrawing (used to denote severe illness requiring admission to hospital in the previous WHO guideline) was 3·2% (95% CI 2·7–3·7). 9968 (83%) of 12 025 children with non-severe pneumonia had at least one risk factor that increased their mortality beyond the upper bound of 95% CI of overall mortality for non-severe pneumonia. Conversely, only one (0·2%, 95% CI 0·004–0·9) of 595 children with non-severe pneumonia and without any of the risk factors analysed died (figure 2; table 4).

## Discussion

We sought to determine the risk factors for pneumonia mortality among children admitted to hospital in settings with high prevalence of comorbidities. By contrast with previous studies in highly controlled research environments,^20^ we proposed to study children who were managed in real-life settings in which most studies on guideline adherence suggest only partial compliance.^21^, ^22^, ^23^, ^24^ This study represents the largest published individual analysis of risk factors for mortality among children with WHO-defined non-severe pneumonia. Pneumonia mortality in our study (5·2%), in a period after the introduction of conjugate vaccines, was higher than mortality reported in a previous systematic review^25^ that included infants younger than 2 months of age (pooled case fatality 3·9%). However, our results are similar to those from a secondary analysis of data from a clinical trial^26^ done in eight Kenyan hospitals before the introduction of the pneumococcal vaccine (overall pneumonia mortality 5·4%). The difference observed between the systematic review and clinical trial^26^ and our study might reflect higher baseline risk in sub-Saharan Africa, or selection of children with low risk in prospective studies included in the systematic review. In our study, less than 0·5% of children with non-severe pneumonia and without any of the risk factors studied died. Mortality among non-severe cases (as defined by WHO) in our study was also higher (2·7%) than other studies that report mortality rates less than 1% for this category of patients.^20^

WHO guidelines recognise severe malnutrition defined by WHZ or mid-upper-arm circumference as a risk factor for mortality, warranting inpatient care when present in association with pneumonia. The use of WAZ is no longer recommended as a screening approach to identify severe acute malnutrition.^4^ We observed a high prevalence of low to very low WAZ (almost 20% of the study population), even after exclusion of cases of severe acute malnutrition were identified by the admitting clinician on the basis of mid upper-arm-circumference, WHZ, visible severe wasting, or oedema due to kwashiorkor. Children with non-severe pneumonia and WAZ less than −3 SD and without severe acute malnutrition had a risk of death almost three times higher than patients with non-severe pneumonia. Increased risk of death among children with poor nutritional status is widely thought to be linked to deficiencies in immune function.^27^, ^28^, ^29^ Consistent with our findings, a systematic review^30^ of children with pneumonia and malnutrition (defined by use of WAZ or WHZ) reported invariably high risks of death for both moderate or severe forms of malnutrition in all 16 studies included.

The presence of pallor, even when classified as mild or moderate, was also strongly associated with mortality across all our analyses. Severe pallor is commonly used as a clinical marker for anaemia in settings where laboratory diagnostics are unavailable, with sensitivity and specificity above 80% for children with packed cell volumes less than 15%.^31^, ^32^ Anaemia is a common presenting feature in sub-Saharan African children, manifesting acutely in conditions such as malaria and chronically because of inadequate nutritional iron intake or helminthic infestation. Severe anaemia has been associated with increased mortality in previous studies of children with pneumonia;^33^, ^34^, ^35^ our study presents new evidence showing risk for mild forms.

These findings have important implications for the WHO policy advocating for outpatient treatment for all but the children with a small set of clinical signs that define severe pneumonia. Although severe pallor would generally warrant admission to hospital, the existing recommendations for case management do not consider very low WAZ or mild to moderate pallor as important risk factors. Thus 1272 (11%) of 12 025 children defined as having non-severe pneumonia in this study with either of these two risk factors would be expected to be managed at home.

A large subpopulation of children had at least one risk factor that was independently associated with mortality, and absolute risks of death higher than the upper confidence limit of that for all non-severe pneumonia cases, but below that for severe pneumonia (mortality between 3% and 10%). The characteristics falling in this category were age younger than 12 months, admission to hospital in a location with high malaria prevalence, elevated respiratory rate (≥70 breaths per min), WAZ less than −2 to −3 SD, lower chest wall indrawing, and moderate dehydration. Our analysis concurs with the findings of a systematic review^36^ of risk factors for mortality in children with pneumonia (almost entirely done in the pre-pneumococcal vaccine period). In this review^36^ that included over 135 000 children from sub-Saharan Africa, the risk factors for mortality in common with our study were low WAZ, female sex, and age younger than 12 months. Infants, who constituted half the population in our study and had mortality three times greater than children aged 12–59 months, are also not assigned priority under the WHO guidelines despite urgent recommendations for review of the guidelines after previous studies.^37^, ^38^

WHO modified the pneumonia case management algorithm to define only two levels of severity in place of the previous three. This recommendation might simplify training of health workers at the primary care and community level by reducing decision making to whether a child requires inpatient or outpatient care. The downgrading of lower chest wall indrawing, previously a sign defining the need for admission, has been challenged in settings of high mortality,^7^ where, among other signs, it is associated with fatal pneumonia.^39^ Our analyses show the presence at hospital level of many children with signs of unrecognised intermediate severity, including lower chest wall indrawing. At least in Kenya, such children appear to have an in-hospital mortality of at least 3%.

Although we highlight associations with mortality in this article, identification of characteristics that might optimise decision making on admission (linked to risk of poor outcome) while being parsimonious to promote simplicity will require additional analyses. In a related article,^40^ we attempt to use machine learning modelling techniques to rank risk factors for pneumonia mortality and use decision curve analysis to explore potential net benefit of the use of highest-ranking risk factors in the decision to admit non-severe pneumonia cases.

Although patients with severe pneumonia were largely prescribed guideline-recommended broad-spectrum antibiotics on admission (unpublished), mortality was more than five times higher than that observed for the non-severe category. The high mortality in this group might suggest late clinical presentation (justifying the need for interventions targeting improved care-seeking or referral structures), or inadequacy of existing treatments, suggesting the need for research to explore alternative antibiotic regimens or interventions to improve supportive care in settings with scarce resources. Supportive care might include improved use of routine pulse oximetry, use of reliable oxygen supplies, appropriate administration of fluids and feeding, and optimisation of nursing care.^41^, ^42^, ^43^

The large sample size taken from multiple hospitals across the country resulted in precise estimates that are broadly representative of the population of children admitted to hospital with pneumonia in many district hospitals in sub-Saharan Africa. The collection of data covering 2 years further enhanced representativeness through elimination of seasonal bias—an important consideration in studies on acute respiratory infections in children.^44^ Our analysis is based on clinical features recorded by many junior clinicians providing routine care; therefore, the interpretation of clinical signs is likely to vary. However, non-differential misclassification resulting from variations in interpretation of signs would be expected to increase random error and reduce the precision of risk estimates. Arguably, inclusion of data captured by many clinicians in routine practice increases the generalisability of our findings.

Analysis is based on documentation of clinical assessments and subsequent data entry. This method has the potential for selection bias arising from misplaced patient records not included in the analysis. However, considerable effort was made to ensure comprehensive sampling through data entry immediately after the end of the inpatient stay with regular comparisons of clinical records retrieved against hospital admission registers. Rigorous training and close supervision of data clerks, use of structured patient admission forms with feedback on their completeness, and dissemination of standard clinical guidelines to health workers at the study hospitals improved the quality of data collected.^45^ We also applied multiple imputation to maximise the use of available data and did sensitivity analyses that supported the assumption that missingness was not associated with severity.

Inadequate individual data on malaria status led us to use hospital location (high *vs* low to very low malaria endemicity) as a crude proxy for this diagnosis. Overlap in clinical presentation of malaria from pneumonia^46^, ^47^, ^48^ might have undermined the validity of risk factor analyses outside high malaria endemic settings. To explore this possibility, we did subanalyses restricted to patients from very low malaria endemic sites (unpublished), which yielded parameter estimates that were consistent with those obtained through use of the full dataset including statistically significant (p<0·0001) associations with mortality for high fever and pallor (two signs common in malaria).

Systematic reviews have shown substantially lower risks of mortality among immunised children than unimmunised children;^30^, ^36^ this association was not apparent in our study. A possible explanation for this absence might be errors of misclassification perhaps attributable to unreliable caregiver-reported data on immunisation status. Inclusion of data on oxygen saturation would have been potentially useful for further definition of risk; however, pulse oximetry is still not commonly available in the study hospitals. Confirmation of the extent to which treatments prescribed such as antibiotics, oxygen, and fluids were received was not possible.

We were unable to collect information on clinical characteristics and outcomes of children with pneumonia managed as outpatients. These data are not available from routine settings in hospitals that contributed data to this analysis, nor any other setting in sub-Saharan Africa. Unobserved factors might have prompted admission of some children—ie, clinicians might have been responding to so-called gut feeling about severity.^49^ Such clinical decisions might have resulted in a higher mortality among children admitted to hospital with a given profile of specified risk factors than among children not admitted to hospital with those same risk factors. However, even if risk of death among patients admitted to hospital with risk factors was higher than the risk among patients not admitted to hospital with the same risk factors, the potential for adverse consequences of implementation of the WHO guidelines in a country such as Kenya cannot be ignored. Thus, discharging of children at high risk would probably result in worse outcomes if hospital admission is effective. The only reason for promulgation of a guideline is to change practice, and in this case, that would mean admission of a reduced proportion of patients shown by our study to have a high risk of death.

Most children admitted to hospital in a period immediately preceding the implementation of policy guidelines that would classify them as having non-severe pneumonia presented with at least one factor associated with a risk of inpatient death above 3%. Among these children, mortality was substantially higher for those presenting with WAZ less than −3 SD or pallor (both mild to moderate and severe) than those without these risk factors. Additionally, infants, admission to hospital in a location with high malaria prevalence, WAZ less than −2 to −3 SD, females, elevated respiratory rate (≥70 breaths per min) or axillary temperature of 39°C or above, lower chest wall indrawing, and some dehydration were independently associated with increased mortality. Our study therefore identifies subgroups of patients at risk for future intervention studies to refine pneumonia case management among inpatients and outpatients. Our findings suggest that presence of WAZ less than −3 SD or any degree of pallor among children with WHO-defined non-severe pneumonia should be considered alongside the WHO criteria for admission care in contemporary African populations—a finding warranting further study. Although this outcome does not constitute formal evidence that making use of these factors would save lives, these results imply a risk of offering outpatient treatment to specific patient subgroups with non-trivial risks of mortality, and provide a foundation for future work to derive a simple risk score for implementation in clinical settings where WHO case management guidelines are in use.
