# Supplementary material for: Appropriateness of clinical severity classification of new WHO childhood pneumonia guidance: a multi-hospital, retrospective, cohort study
Source: Lancet Glob Health. 2017 Dec 12;6(1):e74–83. doi: 10.1016/S2214-109X(17)30448-5 (PMC5732316; doi:10.1016/S2214-109X(17)30448-5)
Supplement: Supplementary appendix [file mmc1.pdf]

THE LANCET
Global Health

Supplementary appendix

This appendix formed part of the original submission and has been peer reviewed. We post it as supplied by the authors.

Supplement to: Agweyu A, Lilford RJ, English M, for the Clinical Information Network Author Group. Appropriateness of clinical severity classification of new WHO childhood pneumonia guidance: a multi-hospital, retrospective, cohort study. *Lancet Glob Health* 2018; **6:** e74–83.

Supplementary Appendix 1: Sensitivity analysis for multiple imputation under missing at random and missing not at random assumptions

A total of 9836/16162 (60.9%) of the observations had missing data for at least one variable. The degree of missingness for individual variables is presented in table 1 of the main article. Multiple imputation was performed for all independent variables included in the analysis which had missing data (immunization status, sex, respiratory rate, temperature, weight for age Z score, pallor and dehydration), as well as the outcome (mortality).

Parameter estimates in the final regression models were fitted with datasets imputed under an assumption of missingness at random (MAR). Departure from this assumption was examined in sensitivity analyses of similar models implemented under the assumption of missingess not at random (MNAR) using pattern mixture models. Sub-groups of patients with similar patterns of completeness of data were defined (no variables missing data, 1-2 variables missing data, and ≥3 variables missing data). The cut-offs of 0 missing variables vs 1-2 missing variables vs ≥3 missing variables were assigned based on the distribution of the degree of missingness across observations to generate three categories of approximately equal size (see table below).

**Missingness category**	**Number of observations**	**Percent**
0 variables missing data	6,326	39.14
1-2 variables missing data	5,792	35.84
≥3 variables missing data	4,044	25.02
**Total**	**16,162**	**100**

Multiple imputation was performed for the three datasets after which associations with mortality for covariates included in the final model were estimated for each sub-group. A summary estimate weighted by the patterns of missingness was then calculated for each covariate, representing the estimate under MNAR. This is presented alongside the analyses implemented assuming MAR in the table below.

Patient characteristic	MI estimates by level of missingness of data			MI estimates weighted by missingness (MNAR assumption)	MI estimates under MAR assumption
	0 variables missing data	1-2 variables missing data	≥3 variables missing data		
	OR (95% CI)	OR (95% CI)	OR (95% CI)	MH OR_95CI	OR (95% CI)
Age 12 - 59 months	ref	ref	ref	ref	ref
Age 2 - 11 months	3.23(2.39-4.37)	2.77(2.13-3.60)	2.64(1.96-3.56)	2.96(2.23-3.92)	2.90(2.46-3.42)
No dehydration	ref	ref	ref	ref	ref
Some dehydration	2.17(1.35-3.50)	1.37(0.82-2.26)	1.31(0.78-2.20)	1.71(1.05-2.80)	1.60(1.20-2.12)
Severe dehydration	3.91(2.63-5.80)	4.39(2.96-6.50)	2.61(1.59-4.29)	4.16(2.80-6.17)	3.64(2.86-4.63)
Temperature <39ºC	ref	ref	ref	ref	ref
Temperature ≥39ºC	1.95(1.46-2.60)	1.59(1.19-2.11)	1.42(1.04-1.94)	1.75(1.32-2.33)	1.66(1.40-1.96)
Low malaria prevalence	ref	ref	ref	ref	ref
High malaria prevalence	1.42(1.06-1.91)	1.05(0.81-1.37)	1.30(0.96-1.77)	1.19(0.91-1.58)	1.24(1.05-1.46)
No pallor	ref	ref	ref	ref	ref
Mild/moderate pallor	3.57(2.55-5.01)	3.34(2.39-4.67)	3.70(2.64-5.20)	3.43(2.45-4.81)	3.58(2.95-4.34)
Severe pallor	6.77(3.85-11.90)	4.45(2.88-6.87)	3.99(1.64-9.75)	5.02(3.12-8.10)	4.72(3.45-6.46)
Non-severe pneumonia	ref	ref	ref	ref	ref
Severe pneumonia	5.12(3.75-6.97)	4.74(3.58-6.26)	3.62(2.75-4.77)	4.90(3.66-6.57)	4.08(3.48-4.79)
Male sex	ref	ref	ref	ref	ref
Female sex	1.54(1.19-2.01)	1.64(1.28-2.10)	1.62(1.23-2.14)	1.60(1.24-2.06)	1.60(1.38-1.86)
Respiratory rate <70 breaths/min	ref	ref	ref	ref	ref
Respiratory rate ≥70 breaths/min	1.45(1.04-2.01)	1.84(1.36-2.48)	1.56(1.12-2.19)	1.65(1.21-2.26)	1.61(1.34-1.94)
Immunization status up to date	ref	ref	ref	ref	ref
Immunization status not up to date	1.35(0.75-2.43)	0.97(0.58-1.61)	1.05(0.59-1.98)	1.12(0.65-1.94)	1.26(0.99-1.81)
WAZ >=-2 SD (none / mild)	ref	ref	ref	ref	ref
WAZ <-2 to -3SD (moderate)	1.76(1.22-2.52)	2.09(1.52-2.86)	2.65(1.90-3.70)	1.94(1.39-2.71)	2.21(1.83-2.68)
WAZ <-3SD (severe)	2.56(1.73-3.77)	1.96(1.32-2.91)	2.63(1.78-3.89)	2.23(1.51-3.30)	2.40(1.92-3.01)

MI – Multiple imputation MAR – missing at random MNAR – missing not at random WAZ – Weight for age Z score

Supplementary Appendix 2: AUC Analyses

Area Under ROC Curve: Complete Case Analysis - All pneumonia Cases

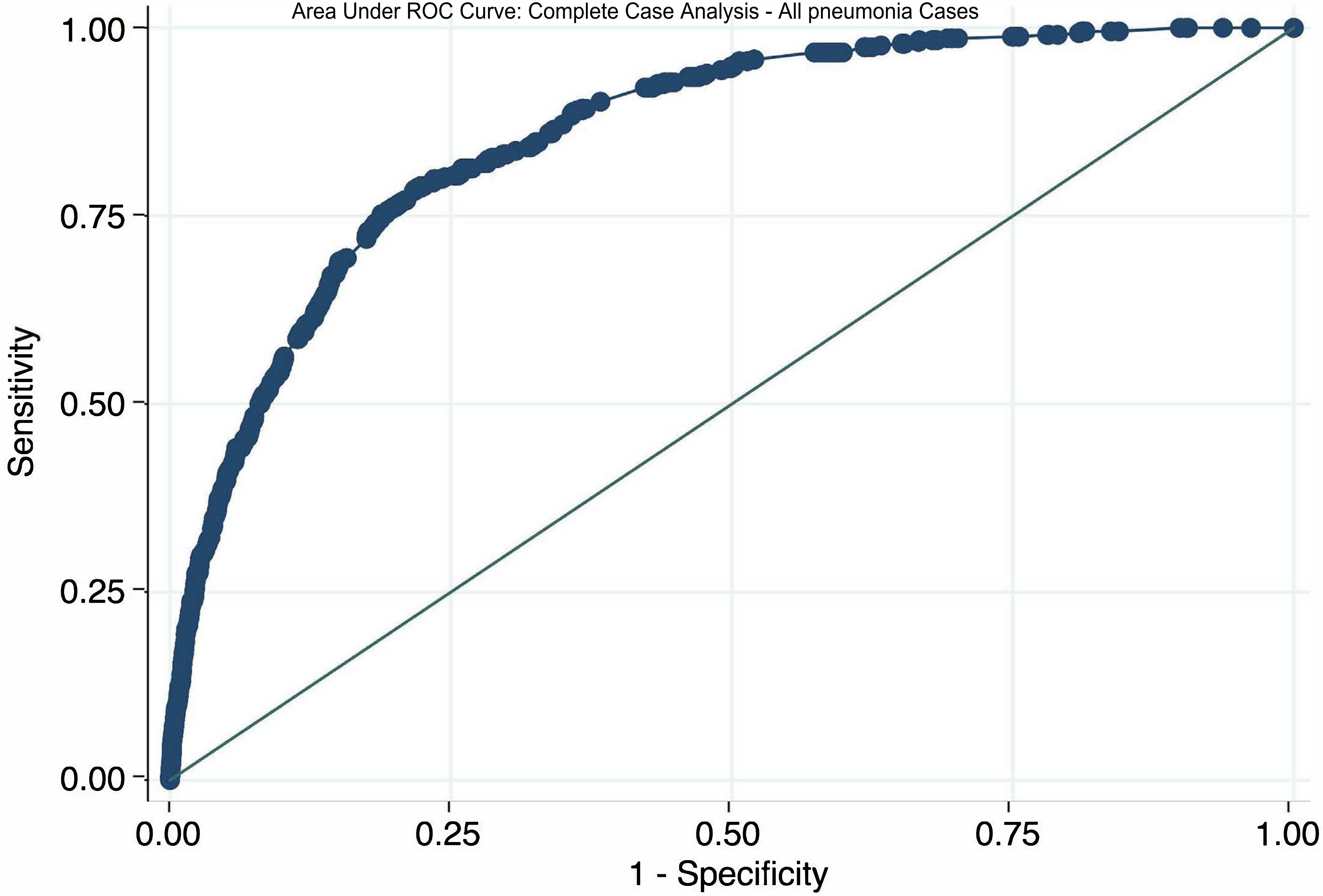

Area under ROC curve = 0.8565

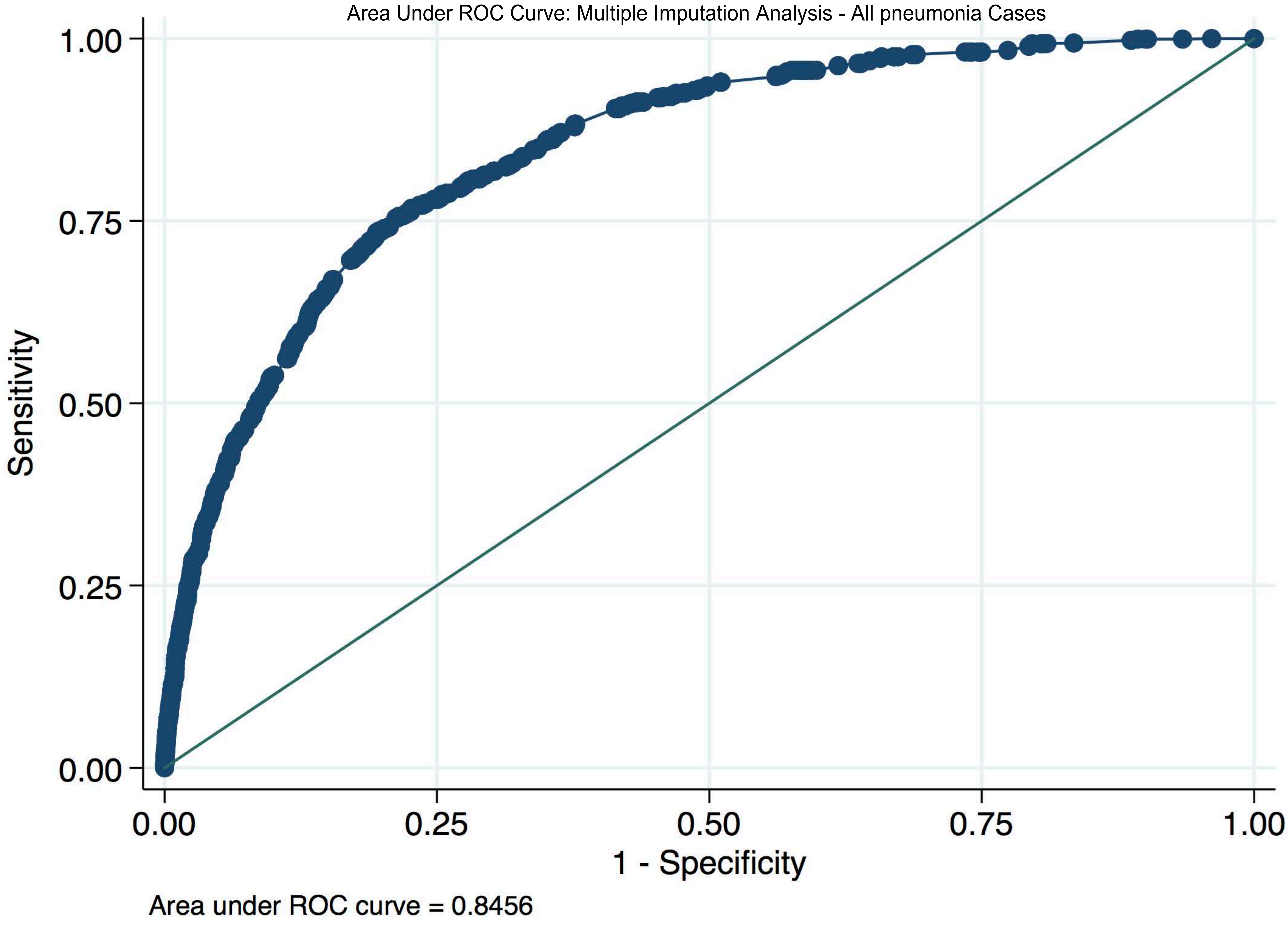
Area Under ROC Curve: Multiple Imputation Analysis - All pneumonia Cases
Sensitivity
1.00
0.75
0.50
0.25
0.00
0.00
0.25
0.50
0.75
1.00
1 - Specificity
Area under ROC curve = 0.8456

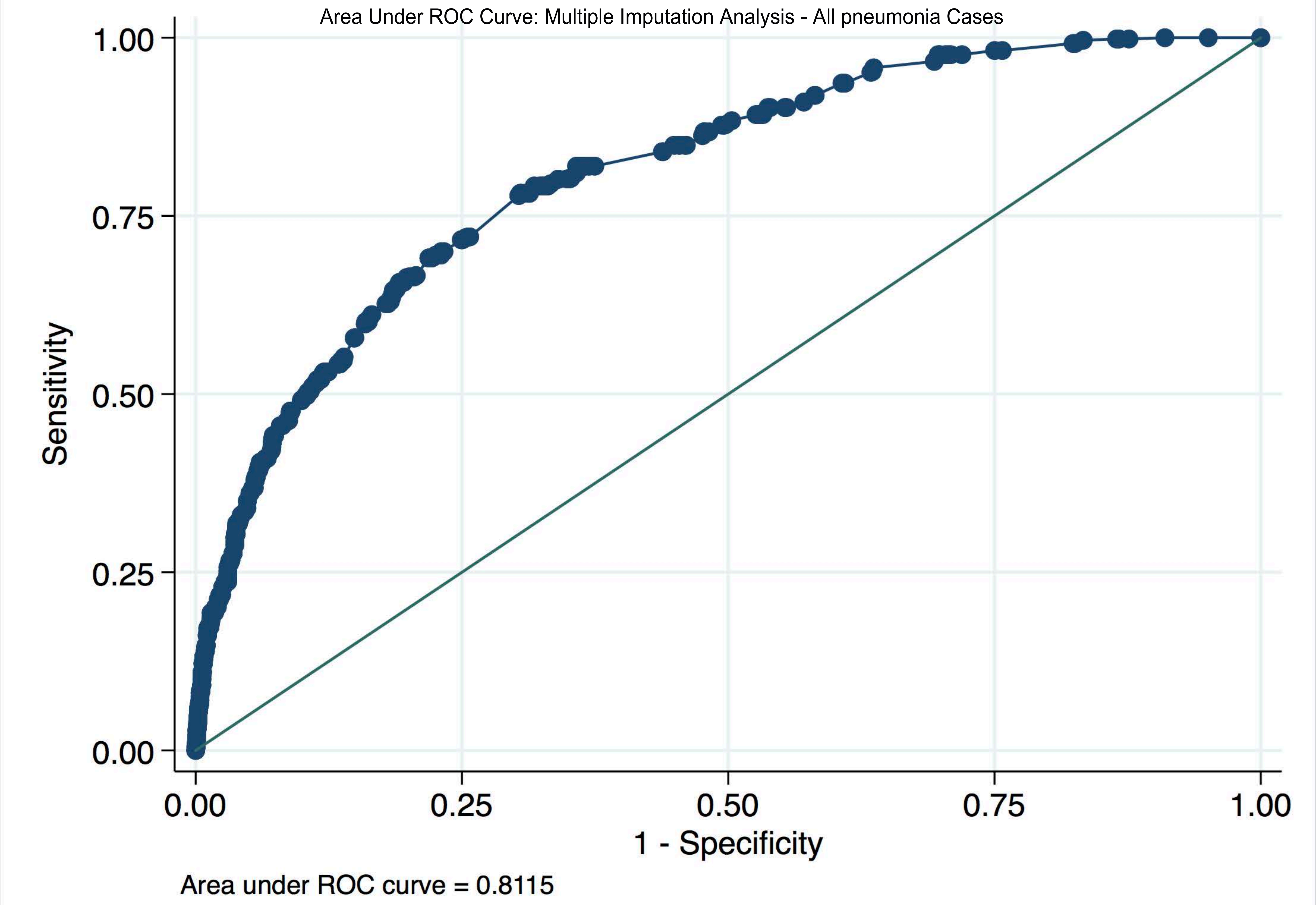
Area Under ROC Curve: Multiple Imputation Analysis - All pneumonia Cases
Sensitivity
1.00
0.75
0.50
0.25
0.00
0.00
0.25
0.50
0.75
1.00
1 - Specificity
Area under ROC curve = 0.8115

Supplementary Appendix 3: Candidate variables

The table below lists variables relevant to this study that were documented for study patients during the initial clinical assessment at the time of admission. The process of selection for inclusion was based on previous literature and clinical plausibility. We also excluded variables that were poorly documented (<50% documentation)

Variable	Documentation (%)
Hospital	16162 (100)
Sex	16011 (99.1)
Age	16162 (100)
Weight	15468 (95.7)
Height	4151 (25.7)
Temperature	11706 (72.4)
Respiratory rate	10992 (68)
Indrawing	12323 (76.3)
Oxygen saturation	4254 (26.3)
Immunization status	9135 (56.5)
Pneumonia severity	15351 (95.0)
Central cyanosis	12332 (76.3)
Grunting	11998 (74.2)
AVPU score	12230 (75.7)
Ability to drink/breastfeed	11717 (72.5)
Pallor	12231 (75.7)
Dehydration	16091 (99.6)
Hemoglobin level	2500 (15.5)
Blood glucose	4273 (26.4)
Malaria slide result	8036 (49.7)
HIV result	6820 (42.2)
Outcome (survival/death)	16031 (99.2)
